# Corrective feedback, individual differences in working memory, and L2 development

**DOI:** 10.3389/fpsyg.2022.811748

**Published:** 2022-12-16

**Authors:** Yi Liao, Wei Zhang

**Affiliations:** ^1^Academy of Arts and Design, Tsinghua University, Beijing, China; ^2^School of Humanities and Communication, University of Sanya, Sanya, China; ^3^Institute of Language Cognition, Carleton University, Ottawa, ON, Canada; ^4^Oriental College of International Trade and Foreign Languages, Haikou University of Economics, Haikou, China

**Keywords:** explicit corrections, meta-linguistic CF, analogy-based CF, English third-person singular form -s, WM

## Abstract

The present study investigated the differential effects of explicit corrections, meta-linguistic corrective feedback (CF), and analogy-based CF on L2 learners' acquisition of English third-person singular form -s and whether and how individual differences in working memory (WM) mediate such effects. One hundred secondary school English-as-a-foreign-language (EFL) learners at a junior middle school in inland China were randomly assigned to the explicit correction group (EG), the meta-linguistic CF group (MG), the analogy-based CF group (AG), and the control group (CG). Learners performed both an information-gap activity and a picture-description activity where their errors on target structure were treated according to their group assignment. The Untimed Grammatical Judgement Test (UGJT) and the Elicited Oral Production Test (EOPT) were used to measure learners' resulting performance. Learners' WM was measured with operation span test. Results revealed that (1) compared to the control group, all the CF groups significantly improved their performance of English third-person singular form -s over time; (2) explicit corrections and meta-linguistic CF displayed superior advantages over analogy-based CF on the immediate posttest. However, the three CF groups demonstrated no significant difference in their performance of English third-person singular form -s on the delayed posttest; (3) WM was only able to predict the effects of analogy-based CF but not explicit corrections and meta-linguistic CF; and (4) analogy-based CF was more favorable to learners with higher WM who can regulate their limited attentional resources more efficiently, whereas explicit corrections and meta-linguistic CF equalize learning opportunities for all learners with different levels of WM. The findings of this study suggest optimal, profile-matched pedagogical options for L2 learning through identifying CF conditions that cater to the needs of young learners with different levels of WM.

## Introduction

Oral corrective feedback (CF) refers to teacher or interlocutor responses to learners' spoken errors (Lyster and Ranta, 1997; Li, 2022). It has been at the forefront of second language (L2) research due to its pedagogical significance (Ellis, 2017). Traditionally, the benefits of CF are extensively examined by exploring the relative effects of exemplar-based CF and rule-based CF, as evidenced by a plethora of empirical studies (Ellis, 2013; Van De Guchte et al., 2015), narrative reviews (Plonsky and Brown, 2015; Nassaji, 2016; Li and Vuono, 2019), and meta-analysis (Li, 2010; Lyster and Saito, 2010). Amidst the evidence of CF in the literature, analogy-based CF, a new exemplar-based form that has been proven to be instrumental to the process and product of L2 learning (Thomas, 2018), receives little attention. As such, analogy-based CF warrants further empirical investigation within the relatively sparse literature by comparing its effects with those traditional ones on the development of L2 knowledge.

Additionally, a host of research shows that the effectiveness of exemplar-based CF and rule-based CF might be correlated with L2 learners' working memory (WM) (Goo, 2012; Mackey and Sachs, 2012; Li, 2013; Yilmaz, 2013; Kim et al., 2015; Fu and Li, 2021). WM, as an important cognitive construct, plays a key role in the allocation and regulation of attentional resources to various dimensions of language (Baddeley, 2015). Although the aforementioned studies could provide a fairly comprehensive picture of the potential role of WM in differential effects of CF, it is imperative to both expand the scope of the investigation and conduct further rigorous research in this area (Li and Zhao, 2021). This could involve looking at CF operationalizations, CF explicitness, self-repair opportunities, as well as various contexts. Critically, no study has examined the mediating role of WM in analogy-based CF along with other traditional ones like explicit corrections and metalinguistic CF; the selection of this CF here could further an understanding of the role of WM in the effectiveness of CF by providing evidence that confirms or disconfirms relevant previous findings. Further, the CF operationalizations and CF explicitness are conflated in previous studies (Li, 2013; Kim et al., 2015), and the manipulation of self-repair opportunities following CF differs from one to another (Goo, 2012; Mackey and Sachs, 2012; Yilmaz, 2013; Fu and Li, 2019, 2021). It is less clear whether these studies evaluating the mediating role of WM in CF are due to differences in CF operationalizations, CF explicitness, or self-repair opportunities following CF. Following this line of thought, investigating WM vis-à-vis CF, where CF explicitness and self-repair opportunities are held constant, would offer a clearer and more nuanced picture of the mechanisms underlying and driving L2 development. Also, most research with respect to the relationship between WM and CF focuses on university-level adults or even older (Mackey and Sachs, 2012; Li, 2013; Kim et al., 2015). The young Chinese English-as-a-foreign-language (EFL) learner context has been hitherto underexplored.

The present study was specially designed to investigate (1) the differential effects of explicit corrections, metalinguistic CF, and analogy-based CF on Chinese EFL learners' acquisition of English third-person singular form -s, and (2) whether and how learners' WM mediates the differential effects of CF.

## Literature review

### Theoretical backgrounds of CF

Theoretical support for the contribution of CF to L2 development draws mainly on the updated Interaction Hypothesis (Long, 2014), the Noticing Hypothesis (Schmidt, 2012), and Skill Acquisition Theory (DeKeyser et al., 2007). According to Long's (2014) hypothesis, CF presents several characteristics: semantic transparency for L2 learners, a contingent juxtaposition of the erroneous forms and target-like models, and unobtrusiveness to the conversational flow. These characteristics can implicitly prime learners' attention to form to induce a cognitive comparison between the erroneous forms and target-like models and, more importantly, encourage form-function mapping for implicit learning. Moreover, the Noticing Hypothesis (Schmidt, 2012) claimed that learners are likelier to learn when they consciously attend to linguistic forms. For this reason, CF increases learners' likelihood of noticing errors and corrections. Additionally, Skill Acquisition Theory (DeKeyser et al., 2007) argues that CF is a guided practice, making it possible to transition L2 knowledge from effortful to more automatic gradually.

Another theoretical issue relevant to the potential roles of CF concerns the new insight on explicit and implicit learning in the field of SLA (Suzuki and DeKeyser, 2017; Godfroid, 2021). This view originates from the two constructs of automatized explicit knowledge and implicit knowledge (Suzuki and DeKeyser, 2017; Godfroid, 2021), which are distinguished by attention to linguistic forms. Using automatized explicit knowledge involves attention to linguistic forms, even if the access is rapid or automatic, whereas using implicit knowledge requires no awareness. Despite the explicit-implicit distinction here, the picture of the interface model suggests that automized explicit knowledge, which develops through explicit learning mechanisms, may impact the acquisition of implicit knowledge (Suzuki and DeKeyser, 2017). With this caveat in mind, the researchers posited that systematic and deliberate CF strategies may lead L2 learners to more correct and rapid use of their knowledge by drawing their attention to linguistic forms (automatized explicit knowledge). After an extended period of practice, learners may not necessarily be aware of their knowledge any longer (implicit knowledge).

With such aforementioned theoretical support, analogy-based CF, in particular, is motivated by analogical learning theory (Kurtz et al., 2001). Kurtz et al. (2001) conceived analogical learning as a process of abstraction by comparing two partially understood systems, and both can be sources and recipients. In the second language acquisition (SLA) context, analogy-based CF focuses on syntactic errors, which requires learners to solve the analogical problem by mapping similar syntactic relations between the analogous and original sentences. It aims to encourage learners to discover the relationship that does not map across productions to find out the source of their error and learn how to correct it by generalizing from the analogy form and applying the abstracted structural relation to their un-target-like utterance in task-based interactions.

While there is general acceptance that CF assists acquisition (Li and Iwashita, 2021; Wang and Li, 2021), previous studies in L2 research have predominately focused on the taxonomy of exemplar-based CF and rule-based CF based on the different underlying learning processes involved (Lai et al., 2020). Exemplar-based CF effectively triggers inductive learning as L2 learners must strive to discover the underlying rule from the CF exemplar. In contrast, rule-based CF effectively triggers deductive learning as L2 learners must test whether the rule holds in the CF exemplar (Thomas, 2018). Providing positive evidence of the target structure makes explicit corrections and analogy-based CF two forms of exemplar-based CF, alongside recasts (Leeman, 2007). Initially introduced in Thomas (2018), analogy-based CF provides a synonymous example in a similar structure to an error in learners' output where the erroneous form is corrected and also includes a guiding question for learners to find and fix the error. For example, if a learner says, “she cook a steak every Friday.” An analogy-based CF might respond: “Almost. If I gave you a similar example, I could say: She makes a steak every Friday. Now can you try that again?” However, instead of providing a correct modal with synonymous items, the explicit correction presents a correct modal with original lexical items. An explicit correction might respond to the preceding example: “Almost. She cooks a steak every Friday.” Unlike exemplar-based forms of CF, meta-linguistic CF is rule-based, presenting underlying rules that are violated (Lyster et al., 2013). Therefore, for the preceding example, a meta-linguistic CF might respond: “Almost. Your subject here is she, so you need a verb form with a third-person singular form.”

### Exemplar-based CF vs. rule-based CF

Many experimental studies have investigated exemplar-based CF and rule-based CF in pairs using a pretest and posttest design, yielding mixed results. No clear advantage was found between exemplar-based CF and rule-based CF in laboratory studies (Lyster and Izquierdo, 2009; Li, 2010). However, in quasi-experimental classroom studies, several studies found that exemplar-based CF were not as effective as rule-based CF (Ellis, 2007; Sheen, 2007; Van De Guchte et al., 2015). Several studies showed no difference between exemplar-based CF and rule-based CF (Loewen and Nabei, 2007; Algarawi, 2011; Thomas, 2018), and other studies found greater effectiveness of exemplar-based CF over rule-based CF (Mifka-Profozic, 2013; Lado et al., 2014).

Based on the theories reviewed herein, explicit claims have been made as to the benefits of analogy-based CF. However, little evidence exists regarding the effectiveness of analogy-based CF in L2 empirical research. To date, Thomas (2018) is the only study that has directly investigated the effects of analogy-based CF in L2 research. She conducted a quasi-experimental classroom-based study with Swedish EFL learners to compare the effectiveness of analogy-based CF with explicit corrections and meta-linguistic CF on the acquisition of English subject-verb agreement. The results demonstrated no clear advantage for the three CF operationalizations. In descriptive statistics indicating different trends over successive testing times, analogy-based CF often led to the lowest performance on the immediate posttest but showed improvement on the delayed posttest.

Apart from CF variations, these inconsistent findings may be attributable to several factors. The first factor concerns the explicitness of CF. Frequently, the variables of CF operationalizations and the explicitness of negative evidence are conflated in studies evaluating the efficacy of CF. This conflation makes it challenging to determine whether the explicitness of negative evidence or the CF operationalizations contribute to L2 learning. Meta-linguistic CF is explicitly corrective, providing explicit negative evidence of an un-target-like structure and containing rule information (Ellis et al., 2006). In contrast, exemplar-based CF usually provides a modal of the target structure but can subsume negative evidence varying explicitness (Li, 2010). Explicit corrections afford explicit evidence, while recasts, which reformulate all or part of the erroneous utterance to correct the erroneous structure, vary from implicit to explicit. The explicitness of recasts depends on a wide range of external and internal factors, including how they are encoded (intonation, stress, number of CF moves, length) (Sheen, 2006; Loewen and Nabei, 2007), developmental readiness (Mackey and Philp, 1998), target structures (Iwashita, 2003; Leeman, 2003; Nakatsukasa, 2021), and the like.

The second factor concerns the self-repair opportunities after receiving CF. Self-repair is a type of uptake in which an error is successfully reformulated. It requires a deeper level of processing and is effective at destabilizing inter-language forms as L2 learners are pushed to reanalyze inter-language representations and attend to the retrieval of alternative forms (Wang and Li, 2021). Swain (1985, 1995) output hypothesis claimed that pushing L2 learners to produce output is indispensable for L2 learners to use their recourses, maximize their linguistic potential, and consider ways of modifying to enhance comprehensibility and intelligibility in their output. Izumi (2002) also suggested that when L2 learners are induced to reproduce their output, they either generate a new message or reprocess their original message, triggering additional grammatical encoding. In Saito (2013) study, the results showed that exemplar-based CF was more effective than rule-based CF. Following the exemplar-based CF + repair procedure, Chinese EFL learners were supplied with ample chances to consider the alternative forms, test their hypotheses about their problematic contrast, and reformulate their L2 knowledge. However, when Chinese EFL learners produced self-repair in response to rule-based CF (i.e., prompts), they needed to create new information to reprocess their original information and develop a rule of target structure contrast on their own to activate their production encoding. Consequently, L2 learners in the rule-based CF group might not possess the ability to reinforce the target-like rule from the subsequent self-repair practice, and this self-repair practice might not be closely associated with their subsequent product development. The third factor concerns the learners' WM, one of the individual cognitive factors. It stands out as clear-cut evidence in clarifying the observed results on the relative efficacy of CF (Goo, 2012; Li, 2013; Yilmaz, 2013).

### WM, CF efficacy, and L2 development

WM, generally defined as “the ability to maintain information in an active and readily accessible state while encouraging and selectively processing new information” (Conway et al., 2007, p. 3), has become a significant focus of cognitively oriented SLA in recent years. The term WM has been adopted for short-term memory to reflect the fact that it is not only a warehouse to store incoming data but also responsible for information processing such as reasoning, general fluid intelligence, language comprehension as well as problem-solving (Baddeley and Hitch, 1974; Just and Carpenter, 1992). The most widely accepted WM model, developed by Baddeley and Hitch (1974), involves a multicomponent memory system composed of a central executive system and other domain-specific subsystems. The central executive coordinates attention control and suppression of irrelevant information, allocating attention, and regulating information processing. The subcomponents include a phonological loop that receives the voice and auditory systems, a visuospatial sketchpad that generates and stores visual information, and an episodic buffer that integrates information from different modalities.

WM is an important cognitive variable influencing L2 learning under oral CF conditions. A great bulk of empirical studies investigating whether and how individual differences in WM are related to the effectiveness of CF have been witnessed in recent years (Granena and Yilmaz, 2018). Within this group, several studies have investigated the associations between WM and a single CF type, such as recasts (Egi et al., 2002; Mackey and Sachs, 2012; Revesz, 2012; Sagarra and Abbuhl, 2013; Li, 2015), or multiple CF strategies such as recasts, metalinguistic CF, or explicit corrections (Goo, 2012; Li, 2013; Mifka-Profozic, 2013; Yilmaz, 2013; Kim et al., 2015; Li et al., 2019). Most studies (Egi et al., 2002; Goo, 2012; Mackey and Sachs, 2012; Revesz, 2012; Li, 2013; Mifka-Profozic, 2013; Sagarra and Abbuhl, 2013; Yilmaz, 2013; Kim et al., 2015; Li et al., 2019) identified significant associations between WM in L2 learning when CF was available. However, the role of WM is not consistent. Some important pieces are still missing. First, variability in WM tests across studies precludes one from concluding the efficacy of CF. In previous studies, simple WM tests include non-word repetition (Egi et al., 2002; Mackey and Sachs, 2012; Revesz, 2012; Zhao, 2015) and digit span (Revesz, 2012), while complex WM tests consist of listening span (Egi et al., 2002; Mackey and Sachs, 2012; Zhao, 2015), aural running span (Kim et al., 2015), reading span (Goo, 2012; Revesz, 2012), speaking span (Mifka-Profozic, 2013) and operation span (Goo, 2012; Yilmaz, 2013). One limitation of the above tests is that learners' WM scores were only based on the recall component of the tests and veracity judgment and did not include reaction time—an important indicator of the processing component of WM (Li, 2013; Fu and Li, 2021). To overcome the limitation here, we employed more synthesized WM tests that recorded the reaction time because WM capacity should involve both the processing and storage functions and because previous studies (Waters and Caplan, 1996; Conlin et al., 2005; Leeser, 2007) showed that there was a processing/storage trade-off. That is, learners sacrificed one component for better performance in another (such as when learners process more slowly in order to achieve more accuracy in word recall). Second, the different operationalizations and explicitness are still conflated in the studies on the mediating role of CF. For example, some studies found that WM only predicted the effects of either exemplar-based CF (i.e., recasts) (Goo, 2012; Sanz et al., 2016; Ahmadian, 2020) or rule-based CF (i.e., metalinguistic CF) (Li, 2013), while other studies found that WM was a significant predictor for both explicit corrections and recasts (Yilmaz, 2013). It remains unclear whether any potential influence of WM on the effectiveness of CF depends on CF operationalizations or the level of explicitness of given CF. Therefore, it is essential to tease out the two distinctions. Third, some studies provide opportunities for learners to modify their output during interaction (Mackey and Sachs, 2012; Li, 2013; Yilmaz, 2013), while others do not (Goo, 2012). Therefore, it is necessary to utilize methodological control to prevent modified output from playing a role as a confounding variable. Finally, there is little evidence regarding the relationship between WM and the effects of analogy-based CF. In general, variations in CF operationalizations, CF explicitness, self-repair opportunities, and WM tests may be confounding variables that interact with WM in influencing L2 learning (Gass and Valmori, 2015).

In English grammar, the formal rule of third-person singular form -s is straightforward. It is conventionally added to the base form of a verb when the subject is he/she/it or an equivalent noun phrase. The literature on the acquisition of English third-person singular form -s has shown that although English third-person singular form -s is highly frequent in input, it is not easily acquired by L2 learners even after many years of exposure to English, especially when learners' L1 lacks the structure. The acquisition of English third-person singular form presents problems to L2 learners because the -s morpheme has low perceptual salience (as they are unstressed) and lack semantic value.

Based on the preceding discussion, the present study thus aimed to empirically examine the relative efficacy of CF operationalizations-explicit corrections, meta-linguistic CF, and analogy-based CF on acquiring English third-person singular form -s, with their explicitness and self-repair opportunities holding constant. In addition, it attempts to use more synthesized WM tests to clarify the potential role of WM in these CF operationalizations, in particular, analogy-based CF, which is largely left unexamined in the Chinese EFL context, to extend the further scope of any possible conclusions in this area. The research questions are as follows:

1. Do explicit corrections, meta-linguistic CF, and analogy-based CF have differential effects on acquiring English third-person singular form -s?
2. How does L2 learners' WM mediate the effectiveness of explicit corrections, meta-linguistic CF, and analogy-based CF?

## Methodology

### Participants

The learners were 103 secondary school students (53 female and 50 male students, aged 11–13 years old) in junior middle school in China. The young learners were selected here based on 2 factors. First, as opposed to adults, most learners in this age group were more likely to make use of CF (Oliver, 2000). Second, such group learners may avoid possible ceiling effects in their learning gains. Before the current research, the learners had learned English for 4 to 9 years (M = 6.2 years). All the learners were native speakers of Chinese, and none had lived or studied in an English-speaking country for more than 1 month. Apart from the time they studied English in class, they had little chance to practice English daily. The learners received a total of 4.5 h of English lessons every week. The English lessons incorporated all skills, including speaking, listening, reading, and writing. In particular, the learners engaged in mandatory English oral classes, which were offered 1.5 h a week by a native English speaker.

The English scores in their mid-term examination (using the standardized Shanghai junior middle school entrance examination) were taken as a reference to ensure that groups were comparable in their L2 proficiency. As a result, three were excluded because they were absent from the mid-term examination. After excluding outliers, the normal distribution of learners' examination scores was verified. 100 learners were randomly divided into four groups: the explicit correction group (EG) (*n* = 25), the meta-linguistic CF group (MG) (*n* = 25), the analogy-based CF group (AG) (*n* = 25) and the control group (CG) (*n* = 25). The one-way ANOVA showed no significant difference across groups, *F*_(3, 96)_ = 1.34, *p* = 0.76. The descriptive statistics are displayed in Table 1.

**Table 1:** Descriptive statistics for proficiency scores.

**EG**			**MG**			**AG**			**CG**		
***n***	**Mean**	**SD**	***n***	**Mean**	**SD**	***n***	**Mean**	**SD**	***n***	**Mean**	**SD**
25	91.16	12.19	25	93.60	7.59	25	98.48	5.66	25	90.64	9.50

EG, the explicit correction group; MG, the meta-linguistic CF group; AG, the analogy-based CF group; CG, the control group.

The untimed grammatical judgment test (UGJT) and the elicited oral production test (EOPT) were designed to measure learners' explicit and implicit knowledge of third-person singular form -s, respectively. The learners' previous knowledge about the target structure was reflected in their pretest scores on the UGJT and the EOPT. To ensure no difference among the four groups in pretest scores, the one-way ANOVA revealed no significant between-group difference in the UGJT: *F*_(3, 72)_ = 0.32, *p* = 0.81, and in the EOPT: *F*_(3, 72)_ = 0.36, *p* = 0.78.

The native English speaker, who was teaching the learners mandatory English oral classes, participated in the study. They were a female native speaker of American English and had a Master of Arts degree. Before data collection, they had 5 years of experience as an EFL instructor at middle schools in China and was willing to assist throughout the study. Before the experiment commenced, the teacher was trained in the provision of CF. First, the teacher was asked to read Lyster et al. (2013) and discuss it with the researcher. Second, the teacher practiced CF with the researcher, and the researcher clarified how they could improve their provision of the assigned CF while keeping their teaching style. Finally, the teacher was required to practice the assigned CF in a different class that was not involved in the present study.

### Target structure

The target structure adopted in the present study is English third-person singular form -s. The consideration for the choice of the target structure is motivated on several grounds.

First of all, Pienemann and Johnston (1987) stated that the learnability of the structure depended on the learner's readiness to acquire it. According to the curriculum of junior middle school, the English third-person singular form -s was chosen because learners were in the process of learning the structure. Second, young learners of 11–13 years old were likely to be familiar with it and had explicit knowledge of this target structure. The purpose here was not to examine whether CF assisted with learning a completely new structure but whether it enabled learners to gain greater control over a structure they have already partially mastered. Third, this target structure introduced in the textbook is not among the morphemes acquired early (Ellis et al., 2006). It is acquired after morphemes such as articles, progressive -ing, and simple-present tense. Although learners with this proficiency would have explicit knowledge of this structure, they sometimes ignore it and make errors in its use. Finally, according to the “Lexical Preference Principle” (VanPatten, 2004), learners naturally rely on the lexical item over the verb inflection so that semantic information can be easily gathered, meaning learners might ignore third-person singular markers as they rely on lexical items to process verbal inflection.

### Treatment tasks

The treatment tasks consisted of four sessions of task-based communicative activities (Information-gap activity, Picture-description activity) to elicit the production of English third-person singular form -s.

In task 1, the information-gap activity was carried out similarly to the one employed in Nakatsukasa (e.g., Nakatsukasa, 2021). It was designed to provide obligatory occasions for using English third-person singular form -s and meets Ellis and Shintani's criteria for a task (e.g., Ellis and Shintani, 2013). Activities were derived from their theme-based textbook (i.e., persons, cities, habits) with a specific linguistic focus in each unit. For the intervention period, the classes focus on a unit called “cities.” Therefore, the teacher divided the learners into small groups and described the task's context “There is a different city, and we need to identify it.” Then some vocabulary words relating to the city's geography, climate, transportation, attractions, and unique features (see Supplementary Table 1) were presented to the learners. After familiarizing these words, each learner was presented with a card. The entire group received a card, “Beijing,” except one received a card, “Guangzhou,” containing the sentences: “You represent the city of Guangzhou, makeup facts so that you will not be found you are different from others.” Each learner was asked to present facts about the city in front of the whole class, based on the card they received. The teacher asked related questions to their presentations, such as—What is the weather like there? How long have you been there?—to increase opportunities for oral production. On average, two related questions were posed to each learner. Also, the spontaneous speech from the learners may contain errors as the activity involved fast online processing. The teacher took this opportunity to provide CF to any utterance containing an English third-person singular form -s (see Example 1). The other learners must listen to one another and adjust their information accordingly so that their facts match those the other group members tell. After presenting their facts about their city, the learners asked one another clarification questions until they found the one who represented the different city.

**Example 1**: Learner: It lie in the northern part of China.
Teacher: Almost. It lies in the northern part of China. Now can you try that again?

In task 2, the picture description task with no time limits was carried out following Sato and Loewen (2018). There were four sets of six pictures, each containing a scenario that created an identifiable activity, such as sitting, painting, or walking in a meaningful context (see Supplementary Figure 1). At the top of each picture was an indication of an order specifying the actions were routine or when these actions took place. Learners were told that they would have 30 seconds to read the pictures and that they needed to describe a person's routine with a list of words according to the meaningful sequence of pictures. They were not allowed to take any written notes. The opening words of the story were given to establish a context for the target structure (i.e., This is Annie. Every year, she celebrates Christmas Eve with her best friends.). If the learners encountered a new word in the activity or temporarily forgot the specific expression of a word, the teacher might provide the correct expression. During the four sessions of task-based activities, the teacher provided explicit corrections, meta-linguistic CF, and analogy-based CF in response to the learners' inappropriate use of English third-person singular form -s. The details are displayed in Table 2.

**Table 2:** Treatment conditions in interactional activities.

**Group**	**Method**	**Example**
EG	It was operationalized to reformulate the learners' nontarget utterances with one change. The full erroneous sentence was recast, emphasizing the verb in the sentence completion to make the corrective force explicit.	S: * It lie in the northern part of China. T: Almost. It lies in the northern part of China. Now can you try that again?
MG	It was operationalized to provide linguistic information to the learners' nontarget utterances. Feedback indicated the number of the subject and the verb.	S: * It lie in the northern part of China. T: Almost. Your subject here is it, so you want a single verb. Now can you try that again?
AG	The initial adverb remained the same along with the preverbal adverb of one was present, a synonymous head noun and modifier were used along with the same determiner (if present), and a synonymous verb was emphasized with intonation.	S: * It lie in the northern part of China. T: Almost. If I gave you a similar example, I could say: It locates in the central part of China. Now can you try that again?
CG	Learners carried out all the interactional tasks but received no CF.	S: * It lie in the northern part of China. T: …, Let's go on.

*Means ungrammatical sentence. EG, explicit correction group; MG, meta-linguistic CF group; AG, analogy-based CF group; CG, control group.

### Tests

A WM test and a series of outcome measures were conducted in the study. The details on the different outcome measures, the number of items and possible points for each measure, and the related estimates of internal validity are presented (see Supplementary Table 2).

#### WM test

The operation span test was adapted from Fu and Li (2021) to measure learners' WM. It shows validity and reliability with young learners in previous L2 studies (Conlin et al., 2005; Li, 2016). During the operation span test, the learners were asked to accomplish 12 testing sets, and the number of items in each set ranged from four to seven. Each testing item consisted of a math question and a letter (i.e., 3^*^7 = 21? C). For each item, the learner looked at the math equation and letter for about 0.2 secs. Then the student made a judgment on the correctness of the equation as quickly as possible while remembering the letter simultaneously. At the end of the testing set, the learners were required to recall and write down the letters shown in the testing set in the correct sequence on paper. At the beginning of the test, learners were familiarized with the testing procedures with three practice sets.

The WM test was conducted in a big language lab, with the teacher in the lab operating the central computer and recording learners' output. The visual stimuli of the WM test were provided via E-prime 2.0, which automatically recorded learners' accuracy and reaction time for each test item. Among four testing spans (four-item, five-item, six-item, and seven-item), the number of test sets was equally distributed, with three test sets for each. Accordingly, there were a total of 66 test items. The maximum score on the WM test was 132, with 66 possible recall points and 66 possible plausibility judgment points.

Three components were involved in the WM test: the number of correct math judgments, the number of correct recalls, and the reaction time to the math judgments. As there was a processing/storage trade-off (Conlin et al., 2005), a composite score was employed in the present study. Namely, higher accuracy and shorter reaction time for math judgments may be achieved at the cost of lower accuracy in letter recall. The raw scores of the three components of the test were transformed to Z scores before averaging the Z scores to achieve a composite WM score. Given that longer reaction time represents lower WM ability, the raw scores for reaction time were multiplied by −1 before being converted to Z scores.

#### Testing instrument

The two tests (the UGTI and the EOPT) were designed to measure learners' explicit and implicit knowledge of English third-person singular form -s, respectively. The UGTI affords measures of explicit knowledge as learners are encouraged to apply rules and have a need to apply metalinguistic knowledge. The EOPT, on the other hand, affords measures of implicit knowledge as learners are guided to use the morphosyntactic target structures while performing a cognitively demanding task (i.e., learners are required to pay equal attention to morphosyntactic, lexical, pragmatic, and phonological aspects of language without much time for accessing their explicit knowledge stored in available memory in a picture description task) (Ellis, 2005). Three separate sets of UGJT and EOPT were prepared. The learners took two tests each time: the EOPT and then the UGJT in that order. The UGJT consists of 20 sentences, and the learners were asked to judge each sentence's grammaticality and correct the ungrammatical sentences. Among the 20 test items, 12 were target items, and 8 were distractors (see Supplementary Data). Regarding the scoring criteria for the UGJT items, if a grammatical sentence was judged to be grammatical, and if an ungrammatical sentence was considered ungrammatical and the error was corrected, 1 point was given. If learners failed to correct the error, no credit was given. Half of the sentences were grammatical, and the other half were ungrammatical. The total mark for each UGJT was 12. Finally, proportions were then calculated.

For the EOPT, the picture description test with no time pressure (Sato and Loewen, 2018) (see Supplementary Figure 2) was used to measure learners' spontaneous use of the target structure. The learners were asked to describe a set of seven pictures, each representing the routines of a group of people. At first, all the characters in the testing materials were introduced with their names on the first picture. The learners were instructed to describe the routines of 2 characters during a regular week. Moreover, they were told to begin their sentences with “Every Monday….” The following seven pictures, including days of the week (Monday through Sunday) and different actions of these two characters, were designed to induce the production of the target structures, with two target structures per picture. For instance, one of the pictures depicted a scene where Camila is drinking coffee while Pablo is playing the guitar. This picture was designed to elicit a sentence like Every Wednesday, Camila drinks coffee, and Pablo plays the guitar on their sofa. Learners were provided a list of words to describe the routines of 2 characters according to the meaningful sequence of pictures. Three sets of pictures were used for the pretests and posttests; the only difference among the three was the different actions taking place on different days.

For the EOPT items, the final scores were calculated as percentages following the formula for Target-Like Use (Pica, 1983). For instance, to compute the exact percentage of English third-person singular form -s that a learner produced in a test, we recorded the numbers for correct usage of English third-person singular form -s in the obligatory contexts where the target structure was required (i.e., *n* correct usage in obligatory contexts), incorrect usage of English third-person singular form -s in the obligatory contexts, and the over-usage of English third-person singular form -s in the contexts where the target structure was not required (i.e., *n* usage in non-obligatory contexts). Then we put the first two numbers together as *n* obligatory contexts and carried the three *n* numbers into the formula below.


$$\begin{array}{l} \frac{n\;correct\;usage\;in\;obligatory\;contexts}{n\;obligatory\;contexts\;+\;over\;usage\;in\;non-obligatory\;contexts}\\ \;\times \;100 \end{array}$$

Two considerations were taken into account when coding English third-person singular form -s. First, when self-correction or repetition occurred, the learner's first production of the verb was counted as one obligatory context and rated. Second, tokens of English third-person singular form -s were tallied; if a learner used the verb “think” twice at different places in their story, then there were two obligatory occasions.

### Procedure

This quasi-experimental study adopted a pretest-posttest design in which data were collected over four sessions in 6 weeks. The first experimental session (held during the last working day of week one) was to complete the WM test. In week 2, session 2 starts with the UGJT pretest and the EOPT pretest, after which the four treatment activities in session three were carried out within four consecutive days. The final instructional treatment was followed by the immediate UGJT posttest and the immediate EOPT posttest. In the fourth session (4 weeks after session 3), the learners took the delayed UGJT posttest and the delayed EOPT posttest (see Table 3). To prevent the influence of the UGJT on the EOPT, the EOPT was always conducted before the UGJT. The number of errors, the number of CF, learners' uptake, and repairs were calculated for further analysis.

**Table 3:** Procedure of the study.

**Session 1**		**Session 2**		**Session 3**		**Session 4**	
**Task**	**Duration**	**Task**	**Duration**	**Task**	**Duration**	**Task**	**Duration**
WM test	20 min	Pretests	40 min	4 treatment activities		Posttest 2	40 min
		• GJT	25 min	• IGA	15 min	• GJT	25 min
		• EOPT	15 min	• PDA	15 min	• EOPT	15 min
				Posttest 1	30 min		
				• GJT	15 min		
				• EOPT	15 min		

WM, working memory; UGJT, untimed grammatical judgement test; EOPT, elicited oral production test; IGA, information-gap activity; PDT, picture-description activity.

## Results

Descriptive statistics for each group were calculated for each test. The normality of the data was assessed by examining whether the values for skewness were less than 1.96. Parametric tests were conducted when the scores were shown to be normally distributed, and non-parametric tests were applied when they were found not normally distributed. However, as the groups of the present study were significantly large, equivalent parametric tests were also conducted to avoid the significant effects of non-parametric tests being underestimated. The results of the parametric tests will only be reported for scores that did not show a normal distribution where they differed from the results of the non-parametric tests.

A one-way ANOVA was carried out on the WM scores of each group to ensure there was no significant difference among the four groups in terms of their WM. Before carrying out the one-way ANOVA, statistical assumptions such as normality and heterogeneity of variances were verified. The results revealed no significant difference, which suggested that the four groups were statistically homogeneous in terms of their WM: *F*_(3, 96)_ = 0.55, *p* = 0.65. Similarly, two one-way ANOVAs on the pretests were conducted concerning the L2 learners' knowledge of English third-person singular form -s. Before carrying out the one-way ANOVA, statistical assumptions such as normality and heterogeneity of variances were verified. The ANOVA did not demonstrate any significant between-group differences, which suggested that the sample pool was homogeneous and that any differences observed in the posttests could be attributed to the treatment: *F*_(3, 72)_ = 0.32, *p* = 0.81, $\eta_{\text{p}}^{2}$ = 0.01, for the UGJT; *F*_(3, 72)_ = 0.36, *p* = 0.78, $\eta_{\text{p}}^{2}$ = 0.02, for the EOPT.

The first research question concerned the differential effects of CF on L2 learners' acquisition of English third-person singular form -s. Results for each UGJT and EOPT are reported separately.

### UGJT

Descriptive statistics for the UGJT are displayed in Table 4. Due to the lack of significant between-group differences in WM score, the decision was made to conduct a 3^*^4 mixed ANOVA. Statistical assumptions such as data normality, Levene tests, and Mauchly test were verified before carrying out the 3^*^4 mixed ANOVA. A mixed 3^*^4 ANOVA with groups as the between-participant variable and times as the within-participant variable revealed that the interaction between time and group was significant, *F*_(6, 144)_ = 14.84, *p* < 0.001, $\eta_{\text{p}}^{2}$ = 0.38. To test whether each group made progress at different time points, we conducted the repeated-measures ANOVA. A set of repeated-measures ANOVAs demonstrated significant effects for time for the EG (*p* < 0.001), the MG (*p* < 0.001), the AG (*p* < 0.001), and the CG (*p* = 0.003). Bonferroni *post-hoc* comparisons revealed significant differences between pretest and immediate posttest for the EG (*p* < 0.001, d = −13.06), the MG (*p* < 0.001, d = −13.00), and the AG (*p* < 0.001, d = −5.23). Also, there were significant differences between pretest and delayed posttest for the EG (*p* < 0.001, d = −5.41), the MG (*p* < 0.001, d = −7.74), the AG (*p* < 0.001, d = −5.05), and the CG (*p* < 0.001, d = −1.825). Finally, there were significant differences between the two posttests for the EG (*p* < 0.001, d = 6.93) and the MG (*p* = 0.008, d = 3.72).

**Table 4:** Means and SDs of UGJT and EOPT scores of the four groups.

			**UGJT**		**EOPT**	
**Tests**	**Group**	***N***	**M**	**SD**	**M**	**SD**
Pretest	EG	25	29.17	17.26	21.95	15.28
	MG	25	25.00	20.83	23.47	14.24
	AG	25	29.17	23.11	18.94	14.98
	CG	25	25.00	22.99	20.84	14.99
Immediate posttest	EG	25	80.17	21.56	68.11	20.21
	MG	25	81.00	22.21	73.87	19.38
	AG	25	55.83	27.64	48.01	19.08
	CG	25	29.00	22.52	23.70	13.89
Delayed posttest	EG	25	51.33	23.25	41.24	19.74
	MG	25	62.50	27.22	49.37	17.97
	AG	25	54.00	25.98	44.97	18.89
	CG	25	33.50	23.58	23.18	15.18

EG, explicit correction group; MG, meta-linguistic CF group; AG, analogy-based CF group; CG, control group.

Two one-way ANOVAs were also carried out to compare posttest scores across groups. The results indicated significant between-group differences among means on the immediate posttest, *F*_(3, 72)_ = 24.06, *p* < 0.001, $\eta_{\text{p}}^{2}$ = 0.50, and on the delayed posttest, *F*_(3, 72)_ = 5.77, *p* = 0.001, $\eta_{\text{p}}^{2}$ = 0.19. Specifically, the effects of the conditions on the immediate posttest were significant in the comparison between the EG and the AG (*p* = 0.03, d = 4.91), between the EG and the CG (*p* < 0.001, d = 11.61), between the MG and the AG (*p* = 0.02, d = 5.02), between the MG and the CG (*p* < 0.001, d = 11.62), and between the AG and the CG (*p* = 0.004, d = 5.32). In addition, the effects of the conditions on the delayed posttest were significant in the comparison between the EG and the CG (*p* = 0.03, d = 3.81) and between the MG and the CG (*p* = 0.005, d = 5.69), and between the MG and the AG (*p* = 0.021, d = 4.13).

### EOPT

Descriptive statistics for the EOPT are displayed in Table 4. Statistical assumptions such as data normality, Levene tests, and Mauchly test were verified before carrying out the 3^*^4 mixed ANOVA. A mixed 3^*^4 ANOVA with groups as the between-participant variable and times as the within-participant variable revealed that the interaction between time and group was significant, *F*_(6, 144)_ = 17.53, *p* < 0.001, $\eta_{\text{p}}^{2}$ = 0.42. A set of repeated-measures ANOVAs demonstrated significant effects for time for the EG (*p* < 0.001), the MG (*p* < 0.001), the AG (*p* < 0.001), and the CG (*p* = 0.001). Bonferroni *post-hoc* comparisons showed significant differences between pretest and immediate posttest for the EG (*p* < 0.001, d = −12.88), the MG (*p* < 0.001, d = −14.82), the AG (*p* < 0.001, d = −8.47), and the CG (*p* = 0.002, d = −0.99). Also, there were significant differences between pretest and delayed posttest for the EG (*p* < 0.001, d = −5.46), the MG (*p* < 0.001, d = −7.99), the AG (*p* < 0.001, d = −7.64), and the CG (*p* < 0.001, d = −0.775). Finally, there were significant differences between the two posttests for the EG (*p* < 0.001, d = 6.73) and the MG (*p* = 0.008, d = 6.55).

As in the case of UGJT, two one-way ANOVAs were also carried out for posttest scores in the EOPT. The results indicated significant between-group differences among means on the immediate posttest, *F*_(3, 72)_ = 39.49, *p* < 0.001, $\eta_{\text{p}}^{2}$ = 0.62, and on the delayed posttest, *F*_(3, 72)_ = 9.44, *p* < 0.001, $\eta_{\text{p}}^{2}$ = 0.28. Specifically, the effects of the conditions on the immediate posttest were significant in the comparison between the EG and the AG (*p* = 0.03, d = 5.11), between the EG and the CG (*p* < 0.001, d = 12.80), between the MG and the AG (*p* = 0.001, d = 6.72), between the MG and the CG (*p* < 0.001, d = 14.88), and between the AG and the CG (*p* = 0.001, d = 7.29). In addition, the effects of the conditions on the delayed posttest were significant in the comparison between the EG and the CG (*p* = 0.004, d = 5.13), between the MG and the CG (*p* < 0.001, d = 7.87), and between the MG and the AG (*p* = 0.003, d = 6.36).

Overall, the results revealed that (1) compared to the control group, all the CF groups significantly improved their performance of English third-person singular form -s over time; (2) explicit corrections and meta-linguistic CF displayed superior advantages over analogy-based CF on the immediate posttest. However, the three CF groups demonstrated no significant difference in their performance of English third-person singular form -s on the delayed posttest.

### CF and the learners' responses

The number of CF and the learners' responses to CF were calculated.

As shown in Table 5, the results for the number of CF in the EG, the MG, and the AG, along with the number of times the CF led to uptake and self-repair, were reported. During the four sessions of task-based communicative activities, a total of 203 numbers of CF were directed to the learners in the EG, and 103 were self-repaired, accounting for 95% of uptake (108 numbers). In the MG, a total of 210 numbers of CF were directed to the learners, and 118 of them were self-repaired, accounting for 92% of uptake (128 numbers). In the AG, a total of 205 numbers of CF were delivered, and 60 of them were self-repaired, accounting for 88% of uptake (53 numbers).

**Table 5:** Descriptive data of CF and learners' responses to CF.

**Group**	**Number of CF**	**Number of uptakes**	**Number of self-repairs**
EG	203	108	103
MG	210	128	118
AG	205	60	53

EG, explicit correction group; MG, meta-linguistic CF group; AG, analogy-based CF group.

The second research question concerned whether and how L2 learners' WM mediates the effectiveness of explicit corrections, meta-linguistic CF, and analogy-based CF. Descriptive analysis with means and standard deviation for the learners' WM scores are presented in Table 6.

**Table 6:** Means and SDs of WM scores of the four groups.

		**Reaction time**		**Math judgement**		**Letter recall**		**ZMW** ^**a**^	
**Group**	***n***	**Mean**	**SD**	**Mean**	**SD**	**Mean**	**SD**	**Mean**	**SD**
EG	25	2871.89	303.99	53.16	6.41	53.24	8.28	−0.14	0.59
MG	25	2789.52	335.25	53.08	7.56	55.2	7.65	0.02	0.61
AG	25	2787.59	375.61	54.24	6.84	54.76	8.43	0.06	0.52
CG	25	2716.26	303.67	53.88	8.11	53.4	7.31	0.06	0.72
All groups	100	2791.32	335.50	53.59	7.28	54.15	7.98	0.00	0.61

EG, explicit correction group; MG, meta-linguistic CF group; AG, analogy-based CF group; CG, control group.

To examine whether and how WM was related to the effectiveness of each CF operationalization on the acquisition of English third-person singular form -s, we carried out simple linear regression analyses with each WM score as a predictor variable and pre-to-post gain score (by subtracting pretest scores from posttest scores) as an outcome variable (see Table 7). Before conducting each simple linear regression analysis, the assumption of linearity was verified; homoscedasticity, normality, and independence were also examined.

**Table 7:** Regression results pertaining to WM and CF.

			**Predictors**		
			**WM**		
**CF**	**Test**	**Timing**	**β**	***p***	**R^2^**
EG	UGJT	Immediate posttest	−0.16	0.45	0.03
		Delayed posttest	0.24	0.25	0.06
	EOPT	Immediate posttest	−0.15	0.47	0.02
		Delayed posttest	−0.17	0.41	0.03
MG	UGJT	Immediate posttest	0.02	0.94	0.00
		Delayed posttest	0.00	0.99	0.00
	EOPT	Immediate posttest	−0.17	0.42	0.03
		Delayed posttest	−0.29	0.15	0.09
AG	UGJT	Immediate posttest	0.79	0.00*	0.62
		Delayed posttest	0.68	0.00*	0.47
	EOPT	Immediate posttest	0.54*	0.01*	0.29
		Delayed posttest	0.42	0.04*	0.18

*p < 0.05; UGJT, untimed grammaticality judgment test; EOPT, elicited oral production test; WM, working memory; β, standardized regression coefficient; R^2^, amount of variance accounted for, EG, explicit correction group; MG, meta-linguistic CF group; AG, analogy-based CF group; CG, control group.

As for the EG, WM was not a significant predictor of the effectiveness of explicit corrections on both immediate and delayed posttests. For the UGJT on the immediate posttest, *F*_(1, 24)_ = 0.59, *p* = 0.45, β = −9.6, R^2^ = 0.03; adjusted R^2^ = −0.02, and on the delayed posttest, *F*_(1, 24)_ = 1.37, *p* = 0.25, β = 11.82, R^2^ = 0.06; adjusted R^2^ = 0.02. For the EOPT on the immediate posttest, *F*_(1, 24)_ = 0.55, *p* = 0.47, β = −6.40, R^2^ = 0.02; adjusted R^2^ = −0.02; and on the delayed posttest, *F*_(1, 24)_ = 0.70, *p* = 0.41, β = −5.35, R^2^ = 0.03; adjusted R^2^ = −0.70.

In terms of the MG, WM was not a significant predictor of the effectiveness of meta-linguistic CF on both immediate and delayed posttests. For the UGJT on the immediate posttest, *F*_(1, 24)_ = 0.01, *p* = 0.94, β = 0.0.73, R^2^ < 0.00; adjusted R^2^ = −0.04, and on the delayed posttest, *F*_(1, 24)_ < 0.00, *p* = 0.99, β = 0.17, R^2^ < 0.00; adjusted R^2^ = −0.04. For the EOPT on the immediate posttest, *F*_(1, 24)_ = 0.42, *p* = 0.42, β = −6.42, R^2^ = 0.03; adjusted R^2^ = −0.01; and on the delayed posttest, *F*_(1, 24)_ = 2.18, *p* = 0.15, β = −10.59, R^2^ = 0.09; adjusted R^2^ = 0.05.

Concerning the AG, WM was a significant predictor of the effectiveness of analogy-based CF on both immediate and delayed posttests. For the UGJT on the immediate posttest, *F*_(1, 24)_ = 37.39, *p* < 0.001,β = 25.83, R^2^ = 0.62; adjusted R^2^ = 0.60, and on the delayed posttest, *F*_(1, 24)_ = 20.24, *p* < 0.001, β = 17.43, R^2^ = 0.47; adjusted R^2^ = 0.45. For the EOPT on the immediate posttest, *F*_(1, 24)_ = 9.58, *p* = 0.005, β = 16.83, R^2^ = 0.29; adjusted R^2^ = 0.26; and on the delayed posttest, *F*_(1, 24)_ = 4.96, *p* = 0.04, β = 0.13.29, R^2^ = 0.18; adjusted R^2^ = 0.14. In addition, as seen in Table 7, the β value suggested that when the learners in the AG had higher WM, the analogy-based CF worked better.

In sum, the results suggested that (1) WM was only able to predict the effects of analogy-based CF but not explicit corrections and meta-linguistic CF; (2) analogy-based CF was more favorable to learners with higher WM who can regulate their limited attentional resources more efficiently, whereas explicit corrections and meta-linguistic CF equalize learning opportunities for all learners with different levels of WM.

## Discussion

### Differential effects of CF

The first question of the present study examined if explicit corrections, meta-linguistic CF, and analogy-based CF have differential effects on L2 learners' acquisition of English third-person singular form -s. The results indicate the following patterns in the UGJT and the EOPT: (1) compared to the control group, all the CF groups significantly improved their performance of English third-person singular form -s over time; (2) explicit corrections and meta-linguistic CF displayed superior advantages over analogy-based CF on the immediate posttest. However, the three CF groups demonstrated no significant difference in their performance of English third-person singular form -s on the delayed posttest.

All three CF operationalizations (explicit corrections, meta-linguistic CF, and analogy-based CF) positively affected the acquisition of English third-person singular form -s. In conjunction with the earlier discussion about the framework of CF, the effectiveness of explicit corrections, meta-linguistic CF, and analogy-based CF can be ascribed to their several facilitating roles with pedagogical purposes.

First, the importance of explicitness via CF cannot be neglected, as the explicit nature of CF makes the corrective intention more salient, which helps the learner's attention shift from meaning to form (Han, 2002; Ellis et al., 2006; Yilmaz and Granena, 2021). In terms of explicit corrections, with the assistance of explicit negative evidence that consisted of a specific signal of un-target-like forms, L2 learners' existing knowledge was cross-examined, and they were guided to notice the gap and look for the appropriate representations. Concerning meta-linguistic CF, by supplying explicit negative evidence of an error and a rule governing usage, L2 learners were trained to concentrate on the inconsistency of their problematic production and their teacher's counterparts so that they were much more sharp-eyed on future instances of linguistic input. As to analogy-based CF, an exemplar-based CF that explicitly provides negative evidence requires deeper processing as the corrected form appears in a different example, thereby increasing the likelihood of noticing. Based on the theoretical support of the Interaction Hypothesis (Long, 2014) and Skill Acquisition Theory (DeKeyser et al., 2007), CF operationalizations with constant explicitness may necessitate three key constructs of L2 learning in general: (1) the word-level noticing of target structures, (2) the restructuring of the existing representations in the interlanguage system, and (3) the establishment and internalization of new representations through practice. Next, the constant opportunities for self-repair that explicit corrections, meta-linguistic CF, and analogy-based CF made available in the present study are another important dimension in explaining the observed learning benefits. The results of the present study revealed that L2 learners generated a high percentage of self-repair after CF. To elaborate, 95%, 92% and 88% after explicit corrections, meta-linguistic CF, and analogy-based CF, respectively. According to Long (2014), this CF-repair sequence could be considered a practice of the learners' output in the context of communicative-oriented interaction. This kind of interaction affords ideal opportunities for acquisition by pushing them to modify their output in the direction of the target language. Another advantage of enhanced self-repair opportunities may originate in its provision of many self-perception opportunities—the more the L2 learners repaired the teacher's CF, the more they perceived their production of English third-person singular form -s. Specifically, in the CF-repair sequence, the many opportunities for self-repair may supply L2 learners with ample chances to consider the alternative forms, test their hypotheses about their problematic contrast, and reformulate their existing knowledge. Consequently, this parameter adjustment assists L2 learners in achieving a more fine-tuned mapping and serves as a strong anchor for improving more target-like output (Saito, 2013).

Compared to explicit corrections and meta-linguistic CF, analogy-based CF tended to result in the lowest means on the immediate posttest and similar means on the delayed posttest. This pattern might be explained by the different depths of processing that different CF operationalizations triggered. Based on the studies reviewed herein, both explicit corrections and meta-linguistic CF provide information more straightforwardly, with explicit corrections providing positive evidence via a model of the target structure and meta-linguistic CF providing a rule governing usage, which involves less retrieval from long-term memory and thus leads to better performance on the immediate posttest. Conversely, according to analogical learning theories (Kurtz et al., 2001), when provided with analogy-based CF, L2 learners must map structures between their erroneous production and the teacher's exemplar to detect and correct their errors. In this regard, they may need time to gradually associate their pre-existing target language knowledge with the information conveyed in analogy-based CF. This pattern might indicate a more significant restructuring of morphosyntactic knowledge for analogy-based CF as “language [that] produced more retrieval from long-term memory and more competition for processing resources may be a better preparation for development to occur” (Lightbown, 2019, p. 37).

### The mediating role of WM

The second question of the present study set out to investigate whether and how WM mediates the effectiveness of CF operationalizations (i.e., explicit corrections, meta-linguistic CF, and analogy-based CF). The results showed that WM could only predict the effects of analogy-based CF but not explicit corrections and meta-linguistic CF. Further, analogy-based CF favored learners with higher WM who can regulate their limited attentional resources more efficiently.

Two possible explanations converged together for the significant role of WM in mediating the effects of analogy-based CF but not explicit corrections and meta-linguistic CF. One possible explanation is that attentional control occurred in the above CF operationalizations, which triggers two different depths of noticing mechanisms. In cognitive psychology and SLA, there is consensus that attentional control is a critical component of WM from the nature of noticing (Baddeley, 2015; Cowan, 2015). Almost all WM models, such as the Multi-Componential Model (Baddeley, 2015), the Executive Attention Model (Engle, 2002), and the Embedded Processing Model (Cowan, 2015), acknowledge the role of attentional control. In the present study, albeit explicitness and self-repair opportunities were kept constant in analogy-based CF, explicit corrections and meta-linguistic CF were more or less straightforward in rejecting the error (i.e., meta-linguistic CF) and presenting the correct form (i.e., explicit corrections). L2 learners in the EG and the MG might have easily noticed the CF operationalizations and quickly used their cognitive resources to infer whether there was an error, its location, and how it should be corrected. In other words, the noticing of explicit corrections and meta-linguistic CF, if it occurred, may not necessarily have been in active operation for them to be noticed. Therefore, attentional control occurred in explicit corrections, and meta-linguistic CF is externally-driven. In stark contrast, analogy-based CF is arguably more attention-demanding as L2 learners are predominately left to their own devices to infer or extract underlying rules in the input (i.e., comparing two analogous utterances to detect similarities and differences, applying the abstract pattern in the CF to the original utterance, and then producing self-repair in response to the induced question). L2 learners' internally-driven attentional control allows them to maintain their erroneous utterances in a readily accessible state while they process meaning and compare their erroneous utterances with the target forms in analogy-based CF.

Another possible explanation for the significant effects of WM in predicting the effects of analogy-based CF is that analogy-based CF could have imposed a heavier online cognitive demand on L2 learners than explicit corrections and meta-linguistic CF due to the need to map the analogous examples in the CF onto the erroneous utterance. Drawing on Skehan (1998) trade-off hypothesis, we could argue that the difficulty of coming up with an analogous example in analogy-based CF on the spur of the moment may impose cognitive pressure on the storage and processing function of WM. Conversely, explicit corrections and meta-linguistic CF impose less cognitive demands on L2 learners. The findings concur with previous findings, which show that WM plays a significant role in cognitive-demanding CF (Goo, 2012; Sanz et al., 2016; Ahmadian, 2020).

The results of the present study revealed that analogy-based CF was more favorable to learners with higher WM. As discussed above, WM was relevant for noticing and pattern identification. We could argue that learners with higher WM can quickly identify patterns and linguistic information from analogy-based CF. This finding and interpretation are in accord with previous aptitude-treatment interaction research, which shows that learners with higher WM tend to notice, identify, register, and process information more efficiently (Yilmaz, 2013; Ahmadian, 2020).

Shifting attention to the present findings of the nonsignificant correlation between WM and the other two CF operationalizations, it is unexpected to find that the result is contradictory to the findings of previous studies, which provide shreds of evidence on the mediating role of WM in metalinguistic CF (Li, 2013) or explicit corrections (Yilmaz, 2013). Closely examining the target structure in Li (2013) and Yilmaz (2013) may offer a plausible reason for the discrepancies here. The target structures of the Chinese qualifier in Li (2013) and the locative structure in Yilmaz (2013) may have been approached by item-based learning. By contrast, the target structure (English third-person singular form -s) provided in the present study requires less cognitive engagement for L2 learners in the EG and the MG. Nonetheless, employing a more comprehensive range of target structures with varying complexity might be worthwhile to determine whether explicit corrections and meta-linguistic CF equalize learning opportunities for all learners with differential levels of WM.

## Conclusion

This study involved a quasi-experimental investigation of the differential effects of explicit corrections, meta-linguistic CF, and analogy-based CF on L2 learners' acquisition of English third-person singular form -s and whether and how individual differences in WM mediate such effects. Results showed that (1) compared to the control group, all the CF groups significantly improved their performance of English third-person singular form -s over time; (2) explicit corrections and meta-linguistic CF displayed superior advantages over analogy-based CF on the immediate posttest. However, the three CF groups demonstrated no significant difference in their performance of English third-person singular form -s on the delayed posttest; (3) WM was only able to predict the effects of analogy-based CF but not explicit corrections and meta-linguistic CF; and (4) analogy-based CF was more favorable to learners with higher WM who can regulate their limited attentional resources more efficiently, whereas explicit corrections and meta-linguistic CF equalize learning opportunities for all learners with different levels of WM. The findings of this study suggest optimal, profile-matched pedagogical options for L2 learning through identifying CF conditions that cater to the needs of young learners with different levels of WM.

Some useful pedagogical implications, albeit tentative, are deducible from these findings. The first implication is that information on the role and taxonomy of CF in L2 development could be provided to all teachers in their in-service and pre-service training. Anyhow, the corrective intention may need to be made clear in specific contexts, such as the context of the present study, so that L2 learners will be encouraged to revise their initial utterances. A second implication is that analogy-based CF, as implemented here, requires deeper processing compared to the other CF operationalizations. Although analogy-based CF showed a facilitative role in this study, L2 learners' knowledge of the target language may affect the effectiveness of analogy-based CF. The learners in this study are middle school students who have been exposed to English for 4–9 years. Based on their L2 knowledge, they can map the analogous exemplars in the CF onto the erroneous sentence and discover the gap. However, analogy-based CF may be of little use for English beginners whose L2 knowledge has not been established. A final implication is that the relationship between WM and L2 development is subject to the subtleties of available CF conditions. For example, analogy-based CF may not be suitable for learners with low WM since it seems lengthier and more complicated than other CF operationalizations. Maybe these are the possible reasons why the teachers in primary school prefer to use short, simple, and didactic recasts (Ha and Murray, 2020).

There are also some limitations in the present study. First, the present study focused on a single target structure. Future studies should include a broader range of structures since the relationship between WM and CF might differ in contexts where CF is provided to other linguistic targets. Second, the participants involved in the present study were considered young learners. Future research should explore whether the same type of WM also mediates the effectiveness of CF in children and adult learners. Third, we did not report any introspective data to determine whether learners were consciously aware of the rules governing the target structure. Future work should be conducted using a stimulated recall paradigm to understand WM's role better. Fourth, as was stated previously, whether teachers receive CF training or not might affect their feedback practices to a certain extent. Therefore, additional future studies are needed to explore this issue.

Despite these limitations, our study contributes to understanding some exemplar-based and rule-based CF operationalizations and the role of WM in mediating their effectiveness. Future research is needed to clarify the effectiveness of CF (these exemplar-based and rule-based CF operationalizations) with (a) different target structures, (b) learners at different proficiency levels, and (c) different introspective measures, such as stimulated recall.

## Data availability statement

The raw data supporting the conclusions of this article will be made available by the authors, without undue reservation.

## Ethics statement

The studies involving human participants were reviewed and approved by University of Sanya Ethics Committee. Written informed consent to participate in this study was provided by the participants' legal guardian/next of kin. Written informed consent was obtained from the individual(s) or minor(s)' legal guardian/next of kin for the publication of any potentially identifiable images or data included in this article.

## Author contributions

YL contributed to the conception of the study and carried out the statistical analysis. WZ drafted the manuscript and provided intellectual content. WZ and YL have reviewed and approved the final manuscript and all agree to be accountable for all aspects of the manuscript and will work together to ensure questions relating to the accuracy and integrity of any part of it are appropriately investigated and resolved. Both authors have read and agreed to the published version of the manuscript.

## Funding

This study was funded by the Ministry of Education, Humanities and Social Sciences, China (No. 20YJC740095), the Provincial Education Department of Hunan, China (No. 20A303), Shanghai Philosophy and Social Science Planning Office, China (No. 2021BYY012), and Hunan Province Philosophy and Social Science Planning Fund Office, China (No. 20YBA163).

## Conflict of interest

The authors declare that the research was conducted in the absence of any commercial or financial relationships that could be construed as a potential conflict of interest.

## Publisher's note

All claims expressed in this article are solely those of the authors and do not necessarily represent those of their affiliated organizations, or those of the publisher, the editors and the reviewers. Any product that may be evaluated in this article, or claim that may be made by its manufacturer, is not guaranteed or endorsed by the publisher.
