# Supplementary material for: Corrective feedback, individual differences in working memory, and L2 development
Source: Front Psychol. 2022 Dec 16;13:811748. doi: 10.3389/fpsyg.2022.811748 (PMC9800285; doi:10.3389/fpsyg.2022.811748)
Supplement: Supplementary file 1 [file Data_Sheet_1.docx]

Supplementary Material

# Supplementary Data

# Untimed grammaticality judgment test (UGJT)

**Instructions:** In this section, you are expected first to decide whether each sentence is grammatically correct or incorrect or not. If the sentence is ungrammatical, please correct it. There is no time control in this section, so you can take your time to double-check your answers.

1） My father **takes** something to read on his way to the office.

A. correct B. incorrect

If ungrammatical, correct it here:

2） * We’re going to the bookstore **in** car this afternoon.

A. correct B. incorrect

If ungrammatical, correct it here:

3）* Everybody **like** Jack because of his honesty.

Grammaticality: A. correct B. incorrect

If ungrammatical, correct it here:

4） She **remembers** exactly everybody’s full name.

Grammaticality: A. correct B. incorrect

If ungrammatical, correct it here:

5) My mother **bought** a picture for me last Sunday.

Grammaticality: A. correct B. incorrect

If ungrammatical, correct it here:

6) * The driver often **remind** passengers to take their belongings.

Grammaticality: A. correct B. incorrect

If ungrammatical, correct it here:

7) The seaside here **attracts** a lot of tourists every summer.

Grammaticality: A. correct B. incorrect

If ungrammatical, correct it here:

8) * My teacher **say** “Good morning” to me yesterday.

Grammaticality: A. correct B. incorrect

If ungrammatical, correct it here:

9) Linda **gets** up at 7 o’clock every morning.

Grammaticality: A. correct B. incorrect

If ungrammatical, correct it here:

10)  * Only you can persuade Alice to **gave** up her foolish idea.

Grammaticality: A. correct B. incorrect

If ungrammatical, correct it here:

11) Green **learns** language by making and correcting mistakes.

Grammaticality: A. correct B. incorrect

If ungrammatical, correct it here:

12) * My grandpa still **treat** me like a child.

Grammaticality: A. correct B. incorrect

If ungrammatical, correct it here:

13) * My wife **walk** to her work place the day before yesterday.

Grammaticality: A. correct B. incorrect

If ungrammatical, correct it here:

14) The house **belongs** to my aunt whom I have never seen before.

Grammaticality: A. correct B. incorrect

If ungrammatical, correct it here:

15) This big shop **offers** more personal service than others.

Grammaticality: A. correct B. incorrect

If ungrammatical, correct it here:

16) * We will have to wait all day unless the doctor **work** faster.

Grammaticality: A. correct B. incorrect

If ungrammatical, correct it here:

17) * John planted two **tree** in the morning and climbed the hill in the evening.

Grammaticality: A. correct B. incorrect

If ungrammatical, correct it here:

18) * Life in the future **would** be much easier.

Grammaticality: A. correct B. incorrect

If ungrammatical, correct it here:

19) It is a common sense that the sun **rises** in the east.

Grammaticality: A. correct B. incorrect

If ungrammatical, correct it here:

20) He **tried** his best but his parents were still not satisfied with him.

Grammaticality: A. correct B. incorrect

If ungrammatical, correct it here:

# Supplementary Figures and Tables

## Supplementary Figures


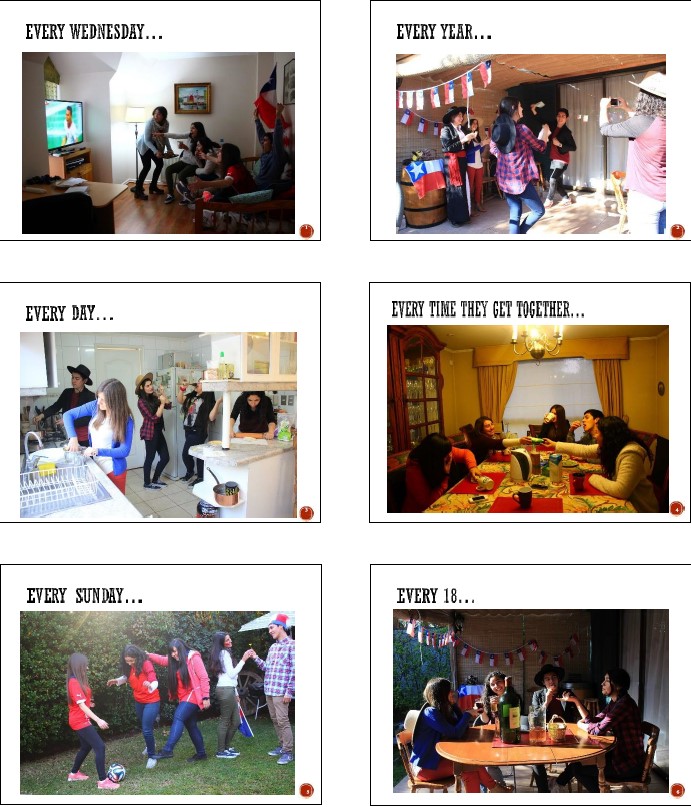


**SUPPLEMENTARY FIGURE 1. Treatment sample for picture description activity**


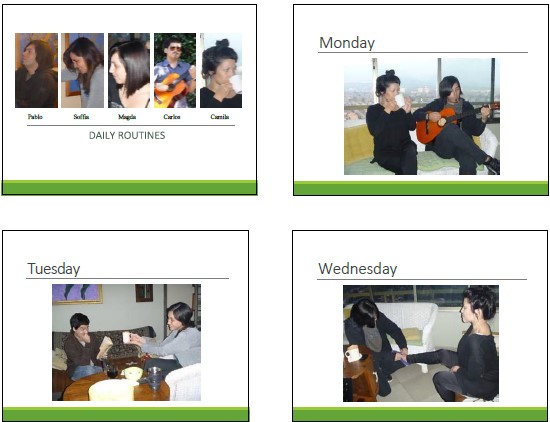


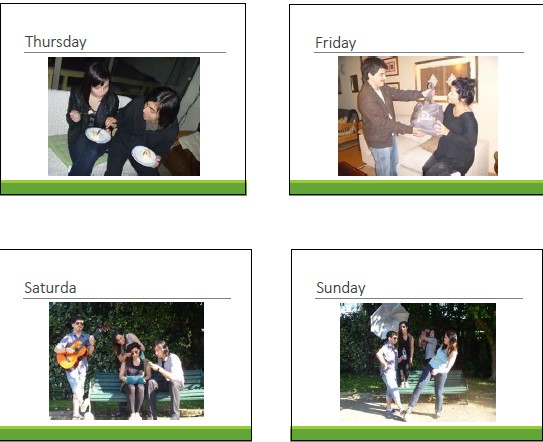


**SUPPLEMENTARY FIGURE 2. Elicited oral picture production test (EOPT)**

## Supplementary Tables

**SUPPLEMENTARY TABLE 1.** **Key words and expressions**

| **Basic Facts** | **Useful Words** |
| --- | --- |
| **Geography** | lie, cover, adjoin, equal, encompass, consist of, border, run through, flow, form |
| **Climate** | change, enjoy, go up to, last, increase, blow, vary, occur, come, rise, decrease |
| **Transportation** | drive, service, provide, connect, remain, enable, run, consist of |
| **Attractions** | remain, reflect, show, boast, contain, attract |
| **Unique Features** | offer, emphasize, serve as, show off, draw, hold, possess, date back, become |

**SUPPLEMENTARY TABLE 2.** **Measures used in the study**

| **Measure** | **Construct** | **Points** | **Reliability** |
| --- | --- | --- | --- |
| MTE  Treatment effect  •UGJT  •EOPT  Operation span test  •Plausibility Judgement  •Recall | Proficiency  Explicit Knowledge  Implicit Knowledge  Working memory | 120  12  TIU  66  66 | .92  .85  .83  .81  .89 |

Note. MTE= middle term examination; UGJT= untimed grammatical judgement test; EOPT= elicited oral production test; TIU= target-like use; Reliability: Cronbach’s а is used as the reliability coefficient; and reliability estimates relating to the UGJT and EOPT are based on the learners’ pretest scores.
